# Head and Neck Squamous Cell Carcinoma Subtypes Based on Immunologic and Hallmark Gene Sets in Tumor and Non-tumor Tissues

**DOI:** 10.3389/fsurg.2022.821600

**Published:** 2022-02-03

**Authors:** Ji Yin, Xinling He, Hui Xia, Lu He, Daiying Li, Lanxin Hu, Sihan Zheng, Yanlin Huang, Sen Li, Wenjian Hu

**Affiliations:** The Affiliated Traditional Chinese Medicine Hospital of Southwest Medical University, Luzhou, China

**Keywords:** head and neck squamous cell carcinoma, immunologic and hallmark gene sets, tumor and non-tumor tissues, prognostic model, bioinformatics analysis

## Abstract

**Background:**

Non-tumor tissue has a significant impact on the prognosis of head and neck squamous cell carcinoma (HNSCC). Previous studies for HNSCC have mainly focused on tumor tissue, greatly neglecting the role of non-tumor tissue. This study aimed to identify HNSCC subtypes and prognostic gene sets based on activity changes of immunologic and hallmark gene sets in tumor and adjacent non-tumor tissues to improve patient prognosis.

**Methods:**

In the study, we used gene set variation analysis (GSVA) to estimate the relative enrichment of gene sets over the sample population, and identified relevant subtypes of HNSCC by Cox regression analysis and the non-negative matrix factorization (NMF) method. The representative gene sets were identified by calculating the differential enrichment score of gene sets between each of the two subgroups, intersecting them, and screening them using univariate Cox regression analysis. The least absolute shrinkage and selection operator (LASSO) regression analysis was used to screen out potential prognostic gene sets and establish a risk model. Finally, genes encompassed in each prognostic gene set were obtained and subjected to enrichment analysis and protein–protein interaction (PPI) in tumor and non-tumor tissues.

**Results:**

We identified three subtypes of HNSCC based on gene sets in tumor and non-tumor tissues, and patients with subtype 1 had a higher survival rate than subtypes 2 and 3. The subtypes were related to the survival status, pathological stage, and T stage of HNSCC patients. In total 450 differentially gene sets and 39 representative gene sets were obtained by calculating the differential enrichment score of gene sets between each of the two subgroups, intersecting them, and screening them using univariate Cox regression analysis. The prognostic model was constructed by LASSO regression analysis, including five prognostic gene sets. Kaplan-Meier analysis indicated that different risk groups and the five prognostic gene sets were associated with survival status in the model. Finally, enrichment analysis and PPI indicated that non-tumor and tumor tissues affect the prognosis of HNSCC patients in different ways.

**Conclusion:**

In conclusion, we provide a novel insight for rational treatment strategies and precise prognostic assessments based on tumor and adjacent non-tumor tissues, suggesting that more emphasis should be placed on changes in adjacent non-tumor and tumor tissues, rather than just the tumor itself.

## Introduction

Head and neck cancer is the sixth most common malignancy worldwide, claiming around 470,000 lives each year (1). Head and neck squamous cell carcinoma (HNSCC) makes up over 90% of all head and neck cancers, with tobacco, alcohol, and human papillomavirus infection being the main risk factors (2). Local recurrence, distant metastases, and treatment failure owing to conventional chemotherapy resistance are the main causes of patient death for HNSCC (3). Among HNSCC patients, 5-year survival rates have barely improved over the past decade and remain below 50% (4). Conclusively, due to the high mortality and poor prognosis of HNSCC, identification of relevant molecular subtypes and prognostic gene sets could enhance patient prognosis through personalized treatment regimens and accurately assessed prognosis.

Previous studies on the classification of HNSCC into different subtypes or prognostic gene sets mainly focused on tumor tissue while greatly neglecting the role of non-tumor tissue. Furthermore, studies of changes in the activity of tumor-related pathways have primarily focused on individual pathways or molecules, rather than systematically examining multiple pathways in tumor and non-tumor samples (5). Zhang et al. constructed three subtypes based on a tumor immune cell infiltration score to characterize the various immune landscapes, which could accurately predict patient prognosis and response to immunotherapy (6). Li et al. identified five ferroptosis-related genes and established a model to predict the prognosis of patients with HNSCC (7). Nevertheless, non-tumor tissue also has a significant impact on the prognosis of tumors (8–11).

Currently, few studies have identified HNSCC subtypes or prognostic gene sets based on activity changes of gene sets in tumor and adjacent non-tumor tissues. Based on the activity changes of immunologic and hallmark gene sets in tumor and non-tumor samples, we identified three clinically relevant subtypes of HNSCC and established a prognostic signature, which included two prognostic gene sets in tumor and three prognostic gene sets in non-tumor samples. Next, genes encompassed in each prognostic gene set were obtained and subjected to enrichment analysis and protein–protein interaction **(**PPI) in tumor and non-tumor tissues, respectively. In conclusion, the study provides a new insight for reasonable treatment regimens and more precise prognostic assessment by indicating that alterations in tumor and adjacent non-tumor tissues should be considered when selecting treatment modalities for HNSCC.

## Materials and Methods

### Preparation of Data

The RNA-seq data of tumor and non-tumor tissues, as well as the corresponding clinicopathological information (TCGA-HNSCC and GSE65858), were downloaded from the The Cancer Genome Atlas database (TCGA: http://portal.gdc.cancer.gov) and Gene Expression Omnibus database (GEO: http://www.ncbi.nlm.nih.gov/geo) (12). Tumor sites included the oral cavity, lip, tonsils, oropharynx, hypopharynx, and larynx for TCGA samples and the oral cavity, oropharynx, hypopharynx, and larynx for GEO samples. Relevant data was extracted by eliminating duplicate data and 0-day follow-up times. In total 4922 immunologic and hallmark gene sets were extracted from Gene Set Enrichment Analysis (GSEA: http://www.gsea-msigdb.org/gsea/index.jsp) (13).

### Identification of HNSCC Subtypes

Gene set variation analysis (GSVA) was used to estimate the relative enrichment of gene sets over the sample population, and the results were displayed in heat map. Cox regression analysis was employed for feature selection and the non-negative matrix factorization (NMF) method was applied to classify samples into several categories (14). We also selected another gene expression profile data set with a different array platform (GSE65858) to validate our classification. The relationship between HNSCC subtypes and clinical characteristics was evaluated by the chi-squared test. To identify representative gene sets, we calculated the differential enrichment score of gene sets between each of the two subgroups, intersected them, and screened them using univariate Cox regression analysis (*p* < 0.05). The above steps were performed through limma, GSEABase, GSVA, heatmap, CancerSubtypes, ggplot2, factoextra, NbClust, VennDiagram, survival and survminer R packages.

### Construction of Prognostic Gene Sets Model

The least absolute shrinkage and selection operator (LASSO) regression analysis was used to screen out potential prognostic gene sets and establish a risk model. To validate the accuracy of the model in predicting survival, Kaplan–Meier analysis was performed to demonstrate the difference in survival between the high- and low- risk groups and validated using the GSE65858 dataset. Survival curves of each prognostic gene set were plotted in the risk model. The R packages used in these operations were limma, glmnet, survival and survminer.

### Exploration of Functional Enrichment and Protein–Protein Interaction

To clarify the mechanism of prognostic gene sets on prognosis, we extracted the genes contained in each gene set and performed the Gene Ontology (GO) and Kyoto Encyclopedia of Genes and Genomes (KEGG) enrichment analysis in tumor and non-tumor tissues, respectively (15). The genes from the N and T gene sets were put separately into STRING (https://string-db.org) to obtain the data on the interaction between genes. PPI networks were obtained with an interaction score greater than 0.7 being retained (16). The PPI network data were imported into Cytoscape v3.8.2, the top three clusters were detected by the MCODE (Molecular Complex Detection) plug-in, and hub genes in each cluster were identified based on degree values (17). These procedures were performed using R packages including org.Hs.eg.db, enrichplot, clusterProfiler, DOSE, ggplot2, colorspace, and stringi.

## Results

### Recognition of HNSCC Distinct Subtypes Based on GSVA

**Figure 1:**
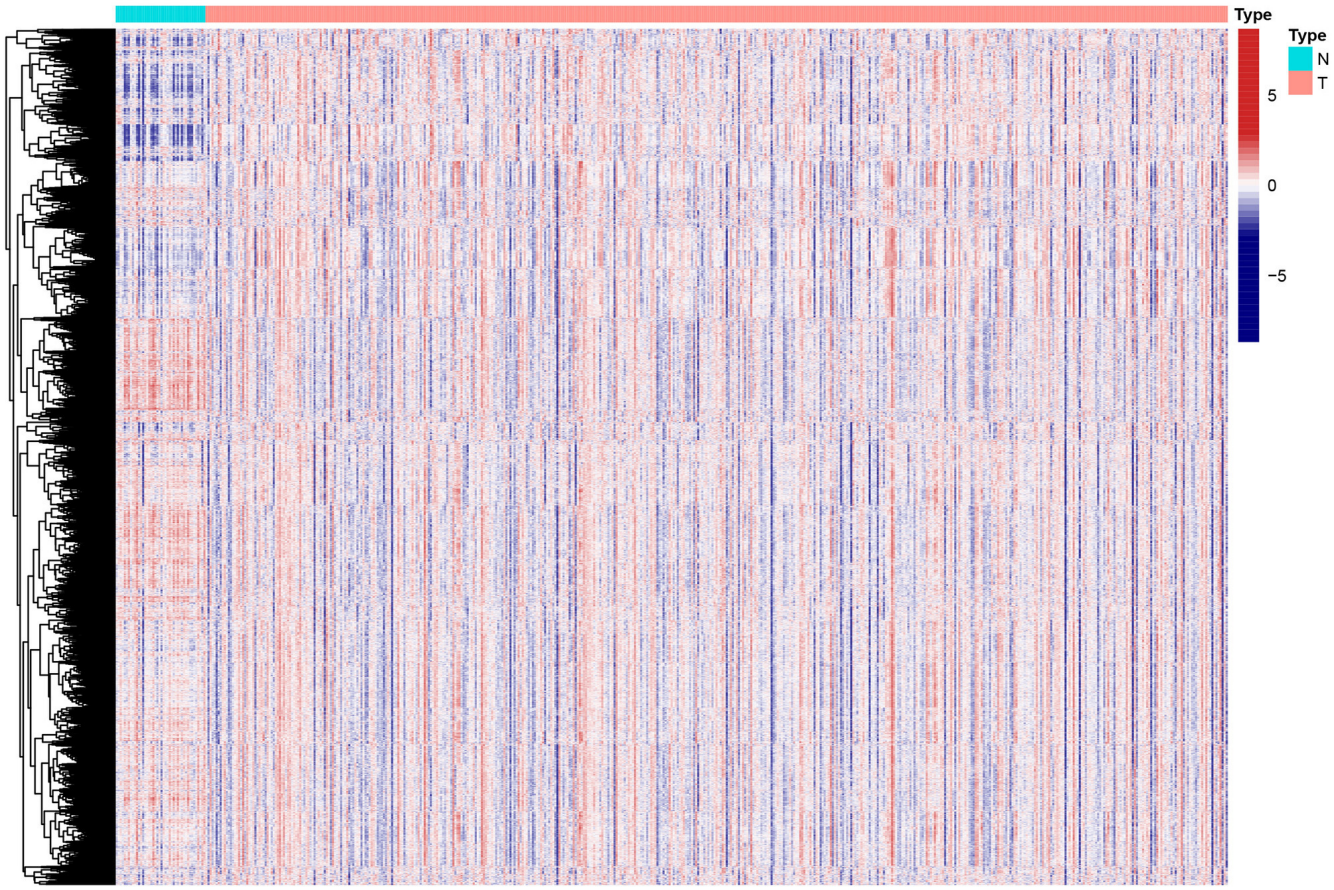
Heat map of enrichment scores from 4922 immunologic and hallmark gene sets in tumor and non-tumor tissues based on TCGA-HNSCC. N, normal; T, tumor.

**Figure 2:**
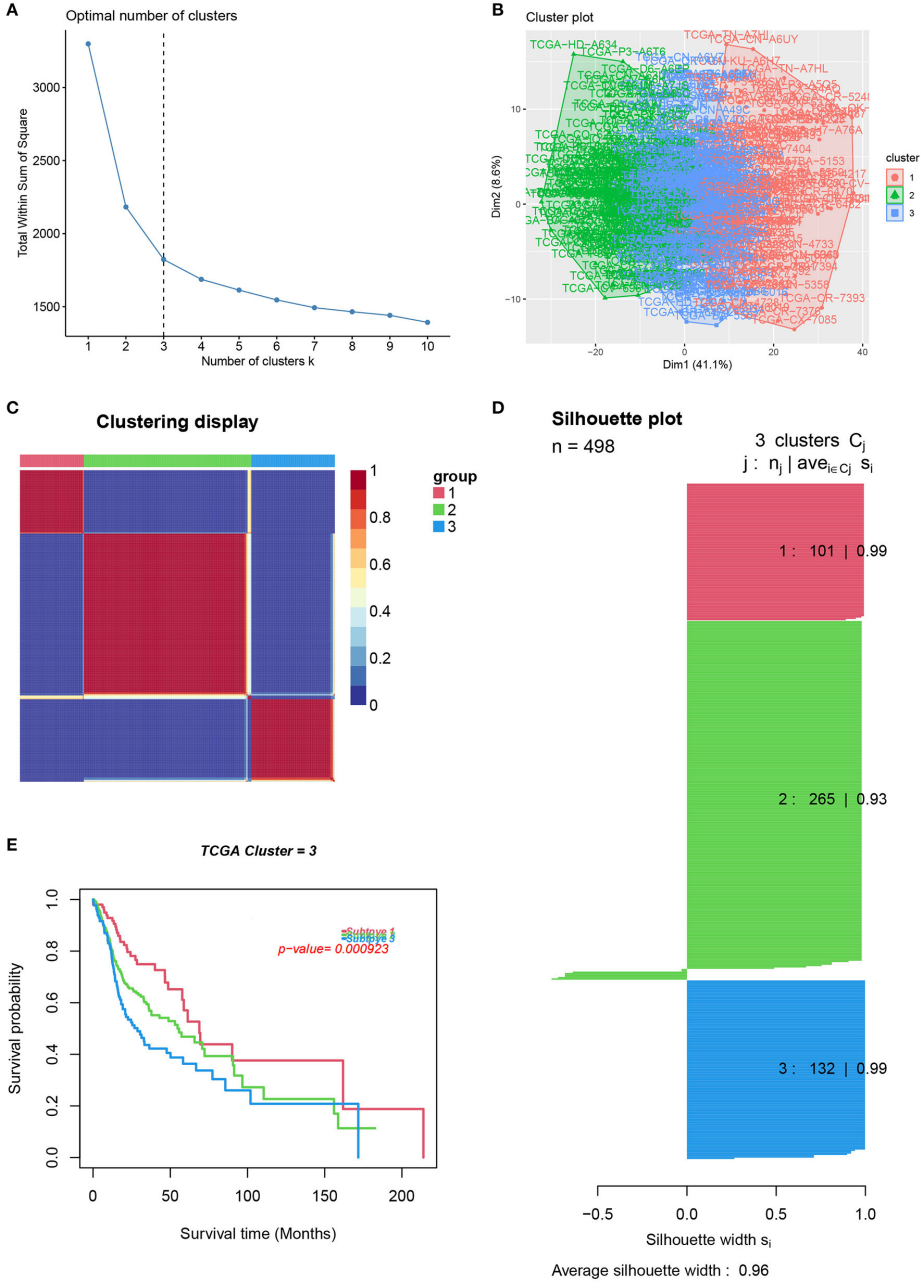
Identification of HNSCC subtypes from TCGA. **(A)** The optimal number of clusters (K) was 3. **(B)** Visualization of cluster results. **(C)** Heat map of NMF clustering results from HNCSS samples with clustering number. **(D)** Silhouette width plots with a value of 0.96. **(E)** Kaplan-Meier survival analysis in different subtypes of HNSCC.

**Figure 3:**
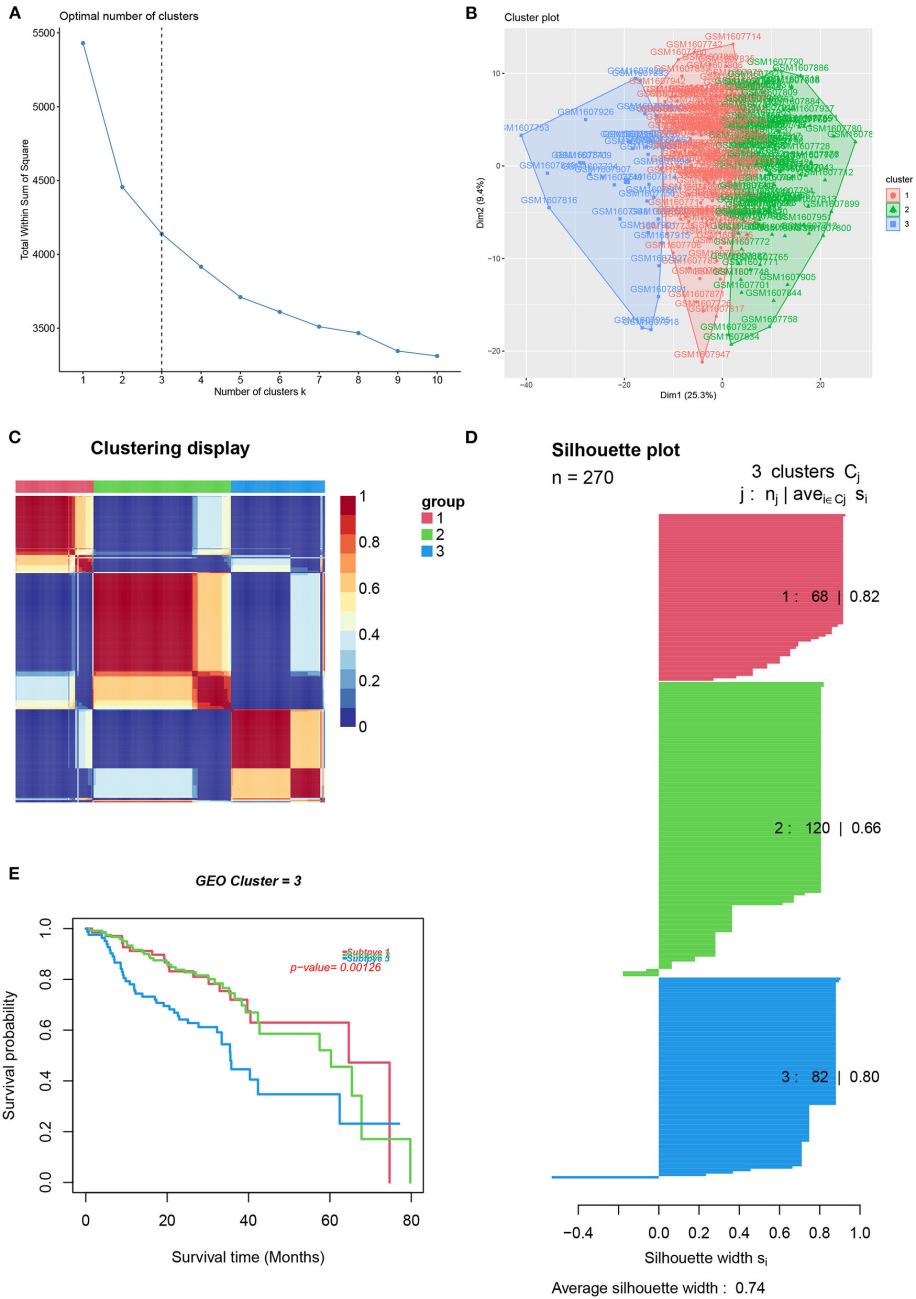
Identification of HNSCC subtypes from GSE65858. **(A)** The optimal number of clusters (K) was 3. **(B)** Visualization of cluster results. **(C)** Heat map of NMF clustering results from HNCSS samples with clustering number. **(D)** Silhouette width plots with a value of 0.74. **(E)** Kaplan-Meier survival analysis in different subtypes of HNSCC.

To reveal the comprehensive spectrum of changes in immunologic and hallmark gene set activity in HNSCC and adjacent non-tumor samples, the enrichment scores of 4,922 gene sets were calculated by GSVA based on the expression profile of TCGA-HNSCC samples; the results are presented in Figure 1. Based on the results of the enrichment scores described above, we attempted to classify HNSCC patients into different subtypes. The results of the NMF method indicated that the optimal number of clusters (K) was 3, HNSCC patients were clustered into three different subtypes, the silhouette width value was 0.96 in silhouette width plots, and HNSCC patients with subtype 1 had a longer overall survival rate compared to types 2 and 3 (Figures 2A–E). In the validation group (GSE65858), the results of the analysis also demonstrated that patients with HNSCC type 1 survived longer, while those with types 2 and 3 survived shorter (Figures 3A–E). The clinical characteristics of different subytpes are shown in Supplementary Table 1. In general, our classification is reasonable and could predict the prognosis of HNSCC patients in different data sets.

### Clinical Evaluation of HNSCC Different Subtypes

**Figure 4:**
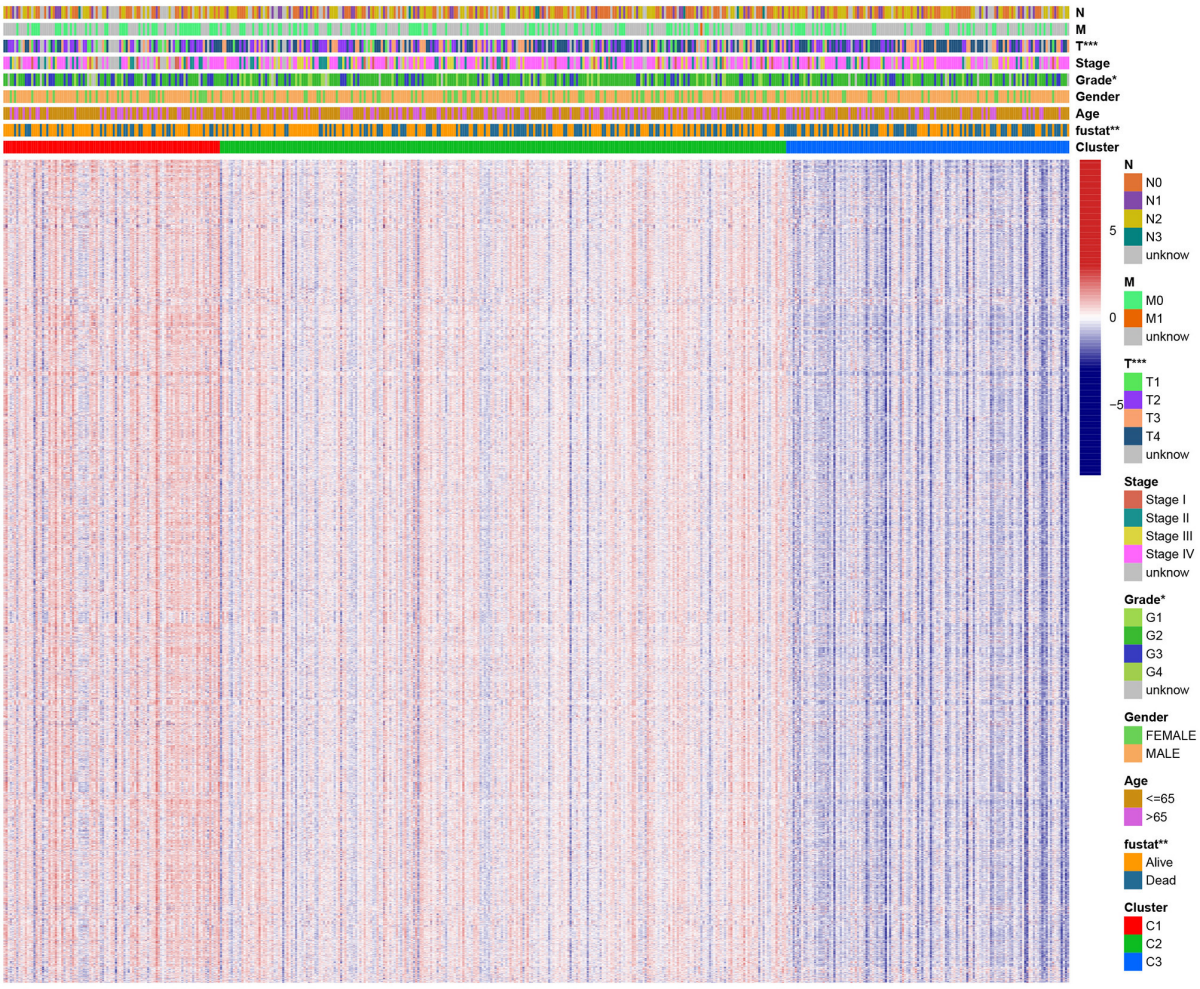
Evaluation of the correlation between clinical characteristics and HNSCC subtypes. The scatter diagram shows that three subtypes were significantly correlated with T stage, pathological stage, and survival status. Heat map of immunologic and hallmark gene sets from different HNSCC subtypes.

**Figure 5:**
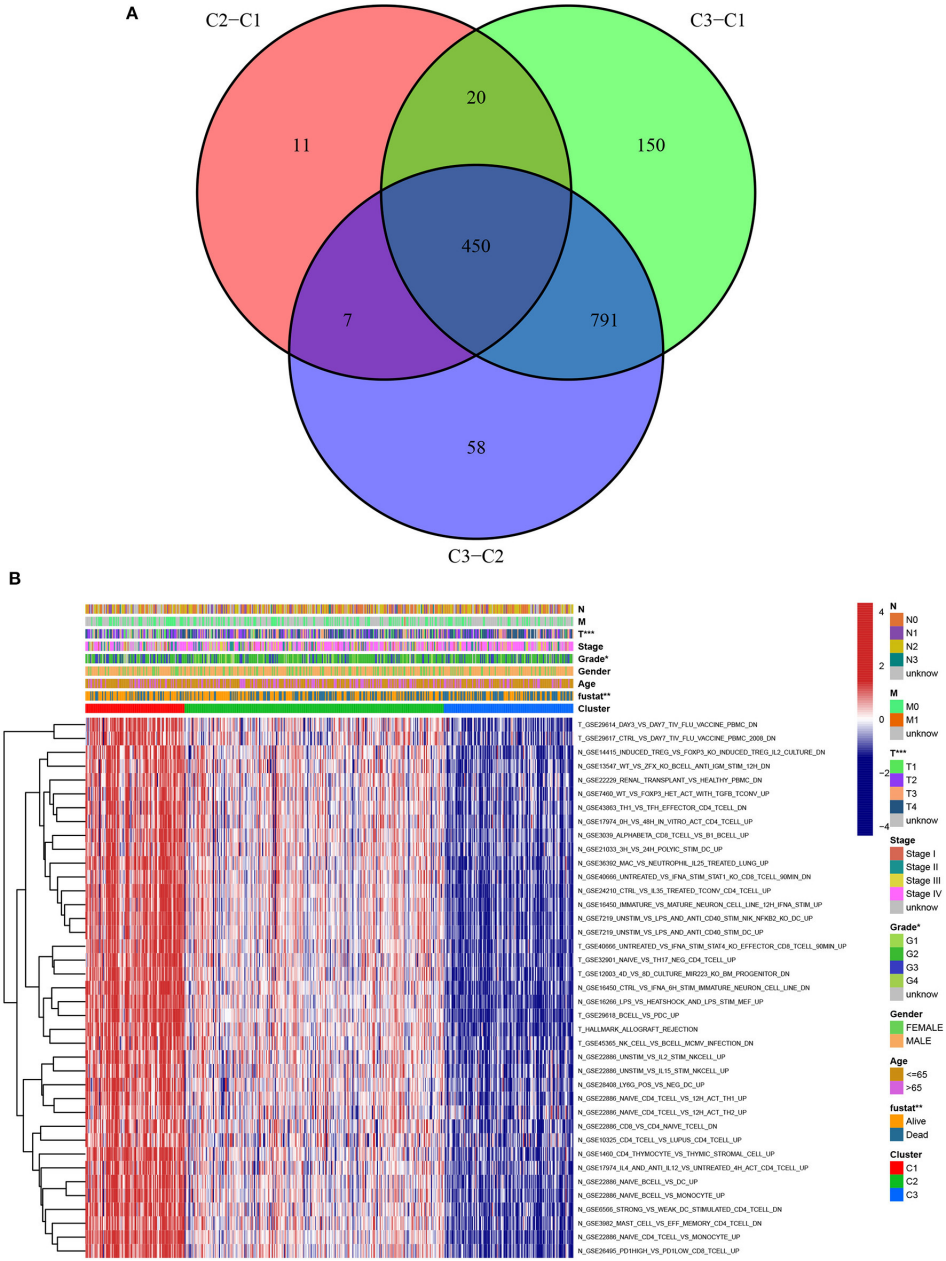
Identification of representative gene sets from HNSCC subtypes. **(A)** 450 differentially gene sets were extracted by the differential enrichment score of gene sets between each of the two subgroups and taking intersections. **(B)** Heat map of 39 representative gene sets in HNSCC subtypes by univariate Cox regression analysis (*p* < 0.05).

A chi-square test was used to investigate the correlation between HNSCC subtypes and clinical characteristics. The strip chart indicated that the subtypes were strongly related to survival status, pathological stage, and T stage of HNSCC patients (Figure 4). To identify representative gene sets, we calculated the differential enrichment score of gene sets between each of the two subgroups, intersected them (Figure 5A), and screened them using univariate Cox regression analysis (*p* < 0.05). We eventually obtained 450 differentially expressed gene sets and 39 representative gene sets. The expression of these 39 representative gene sets in each sample is illustrated in Figure 5B and is detailed in Supplementary Table 2.

### Estimation of Prognostic Risk Assessment Model

**Figure 6:**
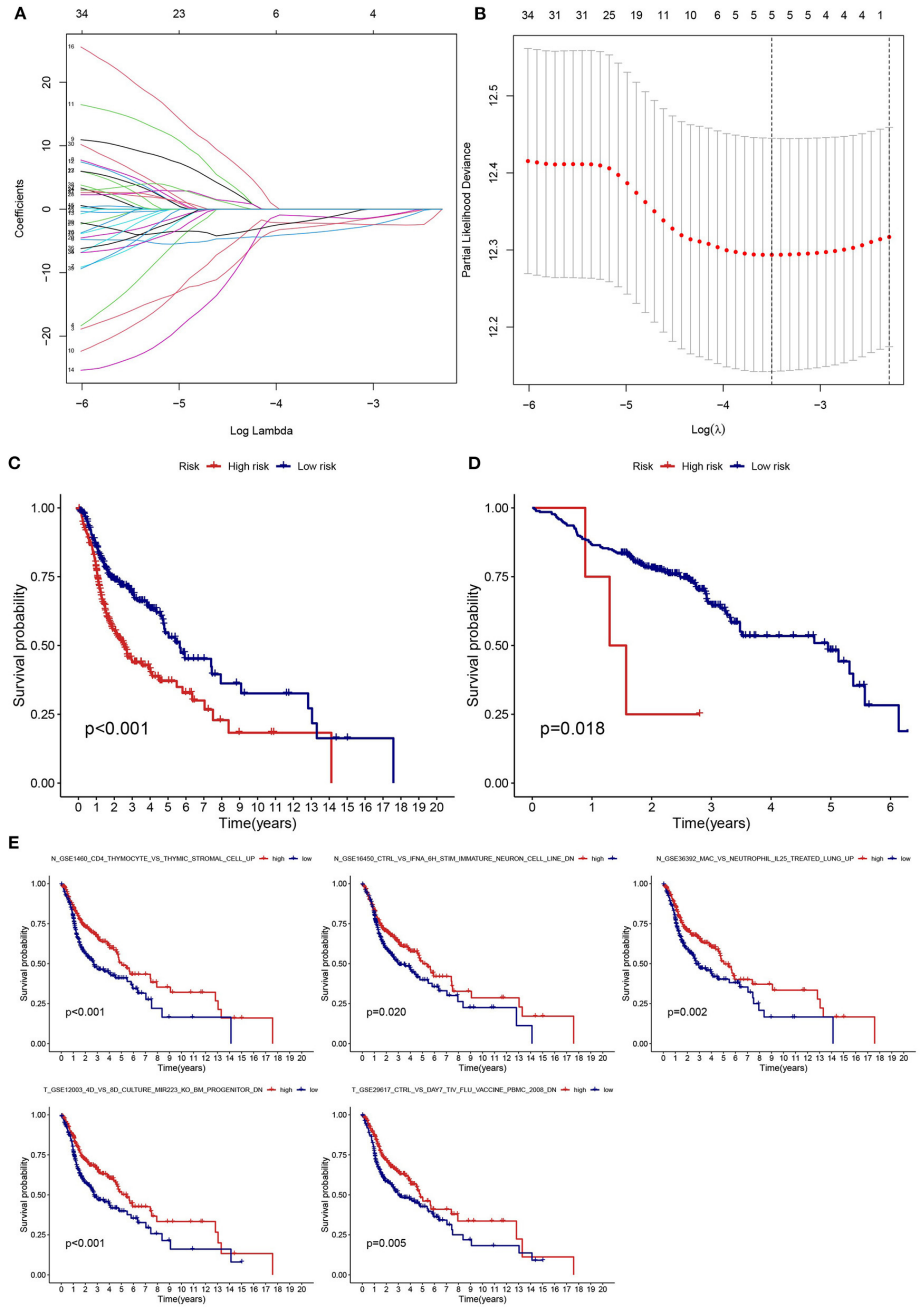
Establishment and validation of a risk assessment model. **(A)** Profiles of LASSO coefficients for five prognostic gene sets. **(B)** Coefficient profile plot generated against the log sequence. **(C,D)** The low-risk group had a longer survival time based on Kaplan-Meier analysis (TCGA and GSE65858). **(E)** Kaplan-Meier survival curves of five prognostic gene sets.

LASSO regression analysis was used to identify prognostic gene sets for HNSCC and construct a prognostic model, including five gene sets (Figures 6A,B). Among these gene sets, three were in non-tumor tissues (N gene sets: N_GSE16450_CTRL_VS_IFNA_6H_STIM_IMMATURE_NEURON_CELL_LINE_DN, N_GSE1460_CD4_THYMOCYTE_VS_THYMIC_STROMAL_CELL_UP and N_GSE36392_MAC_VS_NEUTROPHIL_IL25_TREATED_LUNG_UP), and two were in tumor tissues (T gene sets: T_GSE12003_4D_VS_8D_CULTURE_MIR223_KO_BM_PROGENITOR_DN and T_GSE29617_CTRL_VS_DAY7_TIV_FLU_VACCINE_PBMC_2008_DN). Kaplan-Meier analysis indicated that survival was significantly higher in the low-risk groups (TCGA and GEO) than in the high-risk group (Figures 6C,D). The relationship between the five gene sets and survival status was analyzed, and the results are presented in Figure 6E.

### Investigation of Functional Enrichment and Protein–Protein Interaction

**Figure 7:**
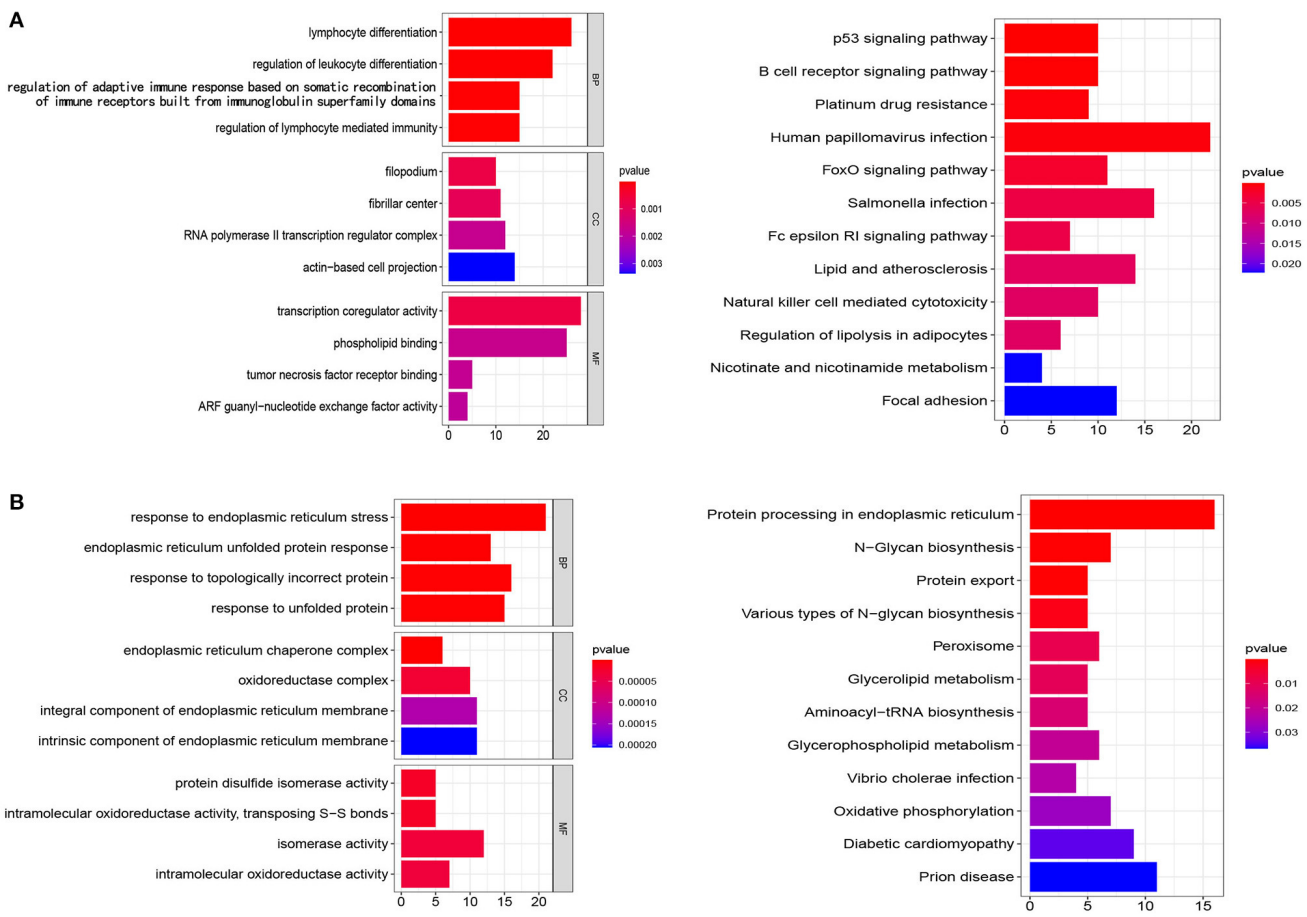
Analysis of GO and KEGG. **(A,B)** The genes from N gene sets and T gene sets were employed for GO and KEGG analysis, respectively. BP, biological processes; CC, cellular components; MF, molecular functions.

To clarify the mechanisms of prognosis for the five prognostic gene sets, we obtained the genes encompassed in each prognostic gene set and performed enrichment analysis and PPI on tumor and non-tumor tissues. In non-tumor tissues, the genes from N gene sets were associated with immune cell regulation, chemotaxis, transcription, and tumor necrosis factor receptor (Figure 7A). The main pathways included the p53 signaling pathway, platinum drug resistance, human papillomavirus infection, FoxO signaling pathway, natural killer cell mediated cytotoxicity, and the B cell receptor signaling pathway (Figure 7A). In tumor samples, the genes from T gene sets were mainly correlated with endoplasmic reticulum, unfolded protein, oxidoreductase, and isomerase (Figure 7B). The main pathways included protein processing and export, N-glycan biosynthesis, glycerolipid and glycerophospholipid metabolism, peroxisome and oxidative phosphorylation (Figure 7B). Supplementary Tables 3, 4 and Supplementary Table 4 detail GO and KEGG enrichment analysis in tumor and non-tumor tissues.

**Figure 8:**
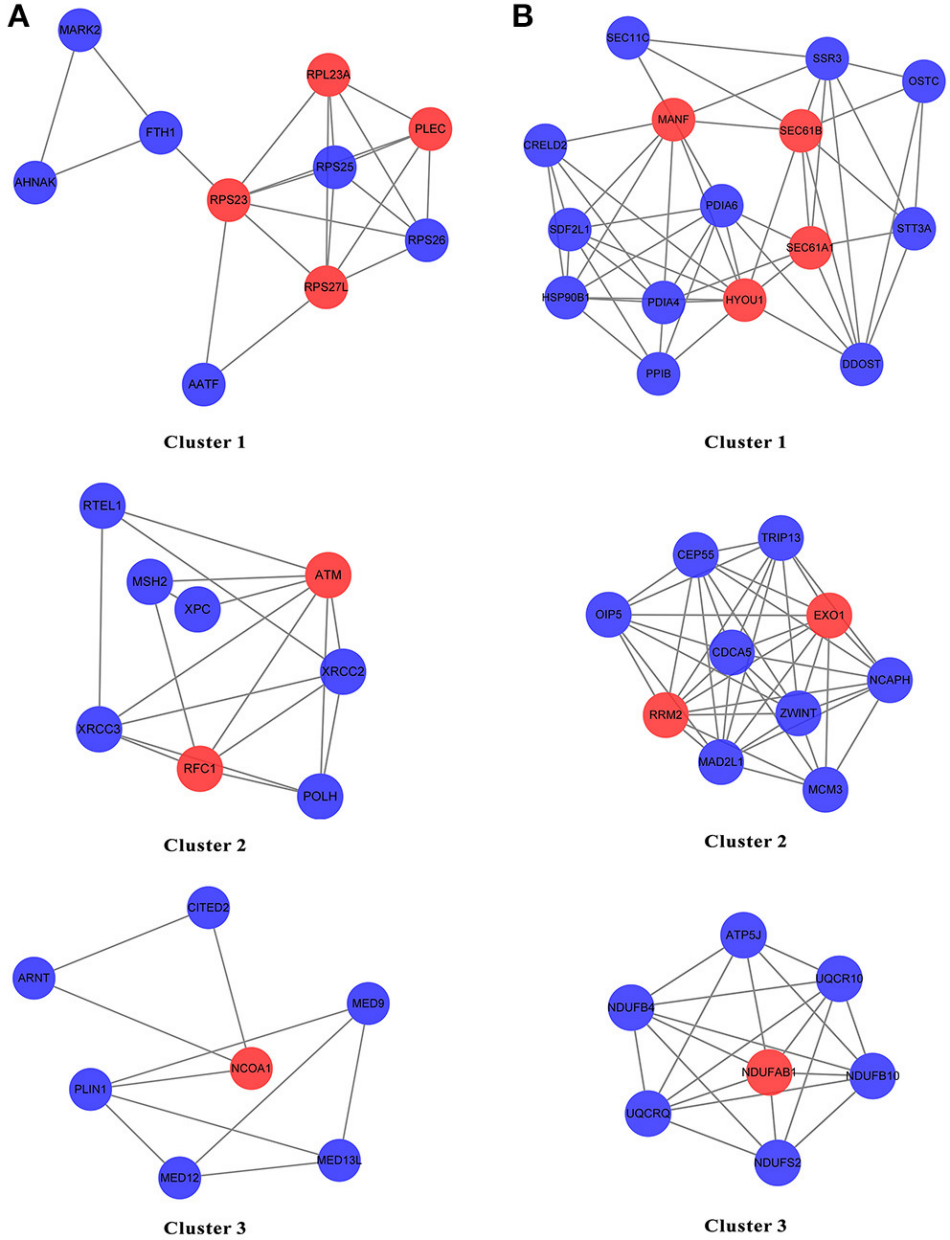
The PPI network of the genes from N gene sets and T gene sets by STRING. **(A,B)** The top three clusters were obtained by the MCODE, and hub genes in each cluster were identified by values of degree.

The genes from N and T gene sets were put individually into STRING to obtain the data on the interaction between genes and PPI networks and scores greater than 0.7 were retained (Supplementary Figures 1, 2). The top three clusters were obtained via the MCODE, and the hub genes in each cluster were identified by values of degree. For N gene sets, the hub genes from cluster 1 were RPS23, RPS27L, RPL23A, and PLEC, cluster 2 were ATM and RFC1, and cluster 3 was NCOA1 (Figure 8A). For T gene sets, the hub genes from cluster 1 were HYOU1, SEC61A1, SEC61B, and MANF, cluster 2 were RRM2 and EXO1, and cluster 3 was NDUFAB1 (Figure 8B).

## Discussion

In this study, we provide a new insight for rational treatment strategies and more precise prognostic assessments based on tumor and adjacent non-tumor tissues, suggesting that more emphasis should be placed on changes in adjacent non-tumor and tumor tissues, rather than just the tumor itself. The leading causes of death in patients with HNSCC include local recurrence and distant metastases (3). After surgical resection of the tumor tissue for HNSCC, the remaining cancer cells may remain viable in the adjacent non-tumor tissue, which is often the cause of local recurrence. Furthermore, HNSCC cells that enter the circulatory system may successfully colonize other tissues through the blood vessels, possibly due to immunological and molecular changes in non-tumor tissues. Therefore, changes in the tumor and adjacent non-tumor tissues should be considered together when choosing therapeutic regimens for HNSCC.

A majority of studies on the classification of HNSCC into different subtypes or prognostic gene sets have focused on tumor tissue and greatly neglected the role of non-tumor tissue. Lee et al. identified three clinically relevant molecular subtypes based on HNSCC tissues that have significantly improved patient prognosis by developing therapies that are tailored to the abnormalities in each patient's cancer cells (18). Zhang et al. identified three subclasses of HNSCC tumors with distinct molecular features and survival outcomes that may contribute to patient stratification and tailored treatment strategies (19). Chen et al. revealed a new immune class in HNSCC with two subtypes characterized by active or exhausted immune responses, tailoring immunotherapeutic strategies for different HNSSC subgroups (20). In our research, we identified three clinically relevant subtypes of HNSCC based on changes of tumor and non-tumor samples, and patients with subtype 1 had a higher survival rate than subtypes 2 and 3.

The results indicated that the five gene sets were highly correlated with the prognosis of HNSCC patients, including three gene sets in non-tumor tissue and two gene sets in tumor tissue. The KEGG pathway analysis of the genes from N gene sets identified various pathways associated with HNSCC, the significant one being the P53 signaling pathway. P53 is a key gene in cellular homeostasis and has been reported to be mutated in a third to two-thirds of HNSCC samples (21). HNSCC patients with P53 mutations have a lower overall survival rate than patients with the P53 wild-type (22). In concert with negatively regulating glycolytic enzymatic activity, P53 curbs glycolysis by down-regulating relevant transporter molecules (23). And, glycolysis plays a key role in HNSCC metabolism, proliferation, migration, and apoptosis (24). However, the main pathways of the genes from T gene sets included protein processing and export, N-glycan biosynthesis, glycerolipid and glycerophospholipid metabolism, peroxisome and oxidative phosphorylation.

Non-tumor and tumor tissues may affect the progression and prognosis of HNSCC in different ways. For non-tumor samples, the genes from N gene sets were associated with immune cell regulation, chemotaxis, transcription, and tumor necrosis factor receptor, which may affect the prognosis of HNSCC patients by regulating immune escape, invasion and migration, and necrosis. In tumor samples, the genes from T gene sets were mainly correlated with endoplasmic reticulum, unfolded protein, oxidoreductase, and isomerase, which may affect the prognosis of HNSCC patients by regulating unfolded protein response and various enzyme-catalyzed reactions. The hub genes for N gene sets included RPS23, RPS27L, RPL23A, PLEC, ATM, RFC1, and NCOA1, while the hub genes for T gene sets included HYOU1, SEC61A1, SEC61B, MANF, RRM2, EXO1, and NDUFAB1. Notably, ATM, RFC1, EXO1, NCOA1, PLEC, and RRM2 have been previously linked to the growth and progression of HNSCC (25–30).

The current study had several shortcomings and limitations. First, as most of the samples from TCGA were non-metastatic, the results may be biased. Second, our total sample size was relatively small, with a disproportionate number of tumor to non-tumor samples (500 tumor and 44 normal samples). Third, the prognostic gene sets identified in our study have not been validated in our clinical sample. In future research work, we plan to continually collect clinical samples and expand sample sizes to validate our model and thoroughly follow up our results.

In conclusion, we have emphasized the prognostic role of non-tumor tissues, indicating that changes in adjacent non-tumor tissues should be considered in the current treatment strategy for HNSCC.

## Data Availability Statement

The datasets presented in this study can be found in online repositories. The names of the repository/repositories and accession number(s) can be found in the article/Supplementary Material.

## Ethics Statement

Ethical review and approval were not required for the study on human participants in accordance with the local legislation and institutional requirements. Written informed consent for participation was not required for this study in accordance with the national legislation and institutional requirements.

## Author Contributions

JY, SL, and WH wrote the manuscript. XH, HX, LHe, and DL performed data extraction and statistical analysis. LHu, SZ, and YH provided guidance and designed figures and tables. SL and WH designed the research. All authors approved the final manuscript.

## Conflict of Interest

The authors declare that the research was conducted in the absence of any commercial or financial relationships that could be construed as a potential conflict of interest.

## Publisher's Note

All claims expressed in this article are solely those of the authors and do not necessarily represent those of their affiliated organizations, or those of the publisher, the editors and the reviewers. Any product that may be evaluated in this article, or claim that may be made by its manufacturer, is not guaranteed or endorsed by the publisher.

## Supplementary Material

Supplementary Figure 1 The PPI network based on the genes from N gene sets.

Supplementary Figure 2 The PPI network based on the genes from T gene sets.

Supplementary Table 1 The correlation between clinical characteristics and subtypes.

Supplementary Table 2 The details of 39 representative gene sets.

Supplementary Table 3 The details of GO enrichment analysis and KEGG pathway analysis based on the genes from N gene sets.

Supplementary Table 4 The details of GO enrichment analysis and KEGG pathway analysis based on the genes from T gene sets.
