# Supplementary material for: Head and Neck Squamous Cell Carcinoma Subtypes Based on Immunologic and Hallmark Gene Sets in Tumor and Non-tumor Tissues
Source: Front Surg. 2022 Feb 3;9:821600. doi: 10.3389/fsurg.2022.821600 (PMC8850349; doi:10.3389/fsurg.2022.821600)

**Supplementary file 1. PRISMA checklist 2020**

| **Section and Topic** | **Item #** | **Checklist item** | **Location where item is reported** |
| --- | --- | --- | --- |
| **TITLE** | | |  |
| Title | 1 | Identify the report as a systematic review. | 1 |
| **ABSTRACT** | | |  |
| Abstract | 2 | See the PRISMA 2020 for Abstracts checklist. | 2 |
| **INTRODUCTION** | | |  |
| Rationale | 3 | Describe the rationale for the review in the context of existing knowledge. | 4-5 |
| Objectives | 4 | Provide an explicit statement of the objective(s) or question(s) the review addresses. | 5 |
| **METHODS** | | |  |
| Eligibility criteria | 5 | Specify the inclusion and exclusion criteria for the review and how studies were grouped for the syntheses. | 6-7 |
| Information sources | 6 | Specify all databases, registers, websites, organisations, reference lists and other sources searched or consulted to identify studies. Specify the date when each source was last searched or consulted. | 7 |
| Search strategy | 7 | Present the full search strategies for all databases, registers and websites, including any filters and limits used. | 7 |
| Selection process | 8 | Specify the methods used to decide whether a study met the inclusion criteria of the review, including how many reviewers screened each record and each report retrieved, whether they worked independently, and if applicable, details of automation tools used in the process. | 7-8 |
| Data collection process | 9 | Specify the methods used to collect data from reports, including how many reviewers collected data from each report, whether they worked independently, any processes for obtaining or confirming data from study investigators, and if applicable, details of automation tools used in the process. | 8 |
| Data items | 10a | List and define all outcomes for which data were sought. Specify whether all results that were compatible with each outcome domain in each study were sought (e.g. for all measures, time points, analyses), and if not, the methods used to decide which results to collect. | 8 |
|  | 10b | List and define all other variables for which data were sought (e.g. participant and intervention characteristics, funding sources). Describe any assumptions made about any missing or unclear information. | Not reported |
| Study risk of bias assessment | 11 | Specify the methods used to assess risk of bias in the included studies, including details of the tool(s) used, how many reviewers assessed each study and whether they worked independently, and if applicable, details of automation tools used in the process. | 8 |
| Effect measures | 12 | Specify for each outcome the effect measure(s) (e.g. risk ratio, mean difference) used in the synthesis or presentation of results. | 8-9 |
| Synthesis methods | 13a | Describe the processes used to decide which studies were eligible for each synthesis (e.g. tabulating the study intervention characteristics and comparing against the planned groups for each synthesis (item #5)). | 8 |
|  | 13b | Describe any methods required to prepare the data for presentation or synthesis, such as handling of missing summary statistics, or data conversions. | 9 |
|  | 13c | Describe any methods used to tabulate or visually display results of individual studies and syntheses. | 9 |
|  | 13d | Describe any methods used to synthesize results and provide a rationale for the choice(s). If meta-analysis was performed, describe the model(s), method(s) to identify the presence and extent of statistical heterogeneity, and software package(s) used. | 9 |
|  | 13e | Describe any methods used to explore possible causes of heterogeneity among study results (e.g. subgroup analysis, meta-regression). | 9 |
|  | 13f | Describe any sensitivity analyses conducted to assess robustness of the synthesized results. | Not reported |
| Reporting bias assessment | 14 | Describe any methods used to assess risk of bias due to missing results in a synthesis (arising from reporting biases). | 9 |
| Certainty assessment | 15 | Describe any methods used to assess certainty (or confidence) in the body of evidence for an outcome. | Not reported |
| **RESULTS** | | |  |
| Study selection | 16a | Describe the results of the search and selection process, from the number of records identified in the search to the number of studies included in the review, ideally using a flow diagram. | 10 |
|  | 16b | Cite studies that might appear to meet the inclusion criteria, but which were excluded, and explain why they were excluded. | Not reported |
| Study characteristics | 17 | Cite each included study and present its characteristics. | Supplementary file 2 |
| Risk of bias in studies | 18 | Present assessments of risk of bias for each included study. | Supplementary file 3 |
| Results of individual studies | 19 | For all outcomes, present, for each study: (a) summary statistics for each group (where appropriate) and (b) an effect estimate and its precision (e.g. confidence/credible interval), ideally using structured tables or plots. | Figure 2 |
| Results of syntheses | 20a | For each synthesis, briefly summarise the characteristics and risk of bias among contributing studies. | 10 |
|  | 20b | Present results of all statistical syntheses conducted. If meta-analysis was done, present for each the summary estimate and its precision (e.g. confidence/credible interval) and measures of statistical heterogeneity. If comparing groups, describe the direction of the effect. | 10-11 |
|  | 20c | Present results of all investigations of possible causes of heterogeneity among study results. | 11 |
|  | 20d | Present results of all sensitivity analyses conducted to assess the robustness of the synthesized results. | Not reported |
| Reporting biases | 21 | Present assessments of risk of bias due to missing results (arising from reporting biases) for each synthesis assessed. | 11 |
| Certainty of evidence | 22 | Present assessments of certainty (or confidence) in the body of evidence for each outcome assessed. | Not reported |
| **DISCUSSION** | | |  |
| Discussion | 23a | Provide a general interpretation of the results in the context of other evidence. | 12 |
|  | 23b | Discuss any limitations of the evidence included in the review. | 13-14 |
|  | 23c | Discuss any limitations of the review processes used. | 14 |
|  | 23d | Discuss implications of the results for practice, policy, and future research. | 14-15 |
| **OTHER INFORMATION** | | |  |
| Registration and protocol | 24a | Provide registration information for the review, including register name and registration number, or state that the review was not registered. | 3 |
|  | 24b | Indicate where the review protocol can be accessed, or state that a protocol was not prepared. | 3 |
|  | 24c | Describe and explain any amendments to information provided at registration or in the protocol. | Not applicable |
| Support | 25 | Describe sources of financial or non-financial support for the review, and the role of the funders or sponsors in the review. | 15 |
| Competing interests | 26 | Declare any competing interests of review authors. | 15 |
| Availability of data, code and other materials | 27 | Report which of the following are publicly available and where they can be found: template data collection forms; data extracted from included studies; data used for all analyses; analytic code; any other materials used in the review. | 15 |

**Supplementary file 2. Summary of the included studies**

| **Study ID** | **Numbers of participants**  **(male: female)** | **Age of the participants (mean or median)** | **Population (condition)** | **Sham acupuncture type** | **Test Acupuncture type** | **Intervention period** | **Time point for outcome assessment** | **Accessed biomarkers** |
| --- | --- | --- | --- | --- | --- | --- | --- | --- |
| Abdi 2012a [1] | A: 83  B: 86 (NR) | A: 38.7, 1.1  B: 37.3, 1.0 | Obesity | (a) | Ear acupuncture | 6 weeks | 6 weeks | FBG, TC, TG, HDL, LDL, CRP |
| Abdi 2012b [2] | A: 82  B: 79 (NR) | A: 37.41, 1.01  B: 36.88, 0.98 | Obesity | (b) | Electroacupuncture | 6 weeks | 6 weeks | FBG, TC, TG, HDL, LDL, CRP |
| Ahsin 2009 [3] | A: 20 (5:15)  B: 20 (7:13) | A: 51.45, 8.9  B: 51.05, 7.73 | Osteoarthritis of knee | (b) | Electroacupuncture | 10 days | 10 days | 𝛽-Endorphin, Cortisol |
| Arnetz 1995 [4] | A: 7  B: 13 | A: NR  B: NR | Environmental illness | (b) | Manual acupuncture | 5 weeks | 5 weeks | Cortisol, NPY |
| Aydoğmuş 2014 [5] | A: 24  B: 28 | A: NR  B: NR | Overactive bladder | (a) | Manual acupuncture | 4 weeks | 4 weeks | NR |
| Bao 2013 [6] | A: 24 (0:24)  B: 23 (0:23) | A: 61 (44-82)  B: 61 (45-85) | Musculoskeletal symptoms in breast cancer patients with aromatase inhibitor | (c) | Manual acupuncture | 8 weeks | 8 weeks | 𝛽-Endorphin, IFN-γ, IL-10, IL-12, IL-1, IL-6, IL-8, IL-17, TNF-α |
| Brustin 2017 [7] | A: 9  B: 11 | Median 40 year | Human papilloma virus-related warts | (a) | Manual acupuncture | 4 weeks | 4 weeks | IL-1, IL-6, IL-8, IL-10, IFN-γ, TNF-α |
| Chassot 2015 [8] | A: 18 (0:18)  B: 16 (0:18) | A: 41.44, 105  B: 39.11, 10.5 | Chronic tension type headache | (a) | Electroacupuncture | 5 weeks | 5 weeks | BDNF |
| Chen 2016 [9] | A: 46 (36:10)  B: 46 (32:14) | A: 56.93, 14.61  B: 55.8/15.75 | Postoperative pain | (a) | Electroacupuncture | 3 days | 3 days | 𝛽-Endorphin, Serotonin |
| Fitri 2019 [10] | A: 20 (2:18)  B: 20 (2:18) | A: 41.5 (20-57)  B: 47 (20-58) | Gastroesophageal reflux disease | (a) | Acupuncture point cat-gut | 15 days | 30 days | IL-6 |
| Haddad-Rodrigues [11] | A: 15 (0:15)  B: 14 (0:14) | A: 28, 33  B: 25, 29 | Anxiety of breast-feeding mothers | (a) | Ear acupuncture | NR | NR | NR |
| Han 2020 [12] | A: 70 (36:34)  B: 70 (37:33) | A: 62.4, 76.7  B: 61.9, 7.9 | Cancer pain | (c) | Electroacupuncture | 2 days | 2 days | Norepinephrine, Serotonin, 𝛽-Endorphin |
| Harbach 2007 [13] | A: 15 (15:0)  B: 15 (15:0) | A, B: 43.4, 11.4 | Chronic low back pain | (b) | Manual acupuncture | 1 day | 1 day | Cortisol, 𝛽-Endorphin |
| Huang 2017 [14] | A: 62 (40:22)  B: 61 (42:19) | A: 58, 10  B: 56, 11 | Asymptomatic hyperuricemia | (c) | Manual acupuncture | 10 days | 1 month | Creatinine, FBS, HbA1C, TC, TG, LDL, HDL |
| Ito 2015 [15] | A: 5 (0:5)  B: 5 (1:4) | A: 35.8, 7.85  B: 42.6, 2.41 | Healthy adults | (a) | Ear acupuncture | 4 weeks | 1 week | Insulin, ACTH, Leptin, Ghrelin, TP, LDL, HDL, FBS, C-peptide |
| Jeon 2015 [16] | A: 7 (0:7)  B: 7 (1:6) | A: 46.3  B: 42.3 | Radioactive iodine-induced anorexia in thyroid cancer patients | (c) | Manual acupuncture | 4 weeks | 4 weeks | ACTH, Cortisol |
| Joos 2004 [17] | A: 24 (7:17)  B: 27 (8:19) | A: 36.2, 8.8/ B: 39.9, 7.7 | Crohn’s disease | (b) | Manual acupuncture | 4 weeks | 4 weeks | A1-glycoprotein, CRP |
| Jubb 2008 [18] | A: 34 (8:26)  B: 34 (5:29) | A: 66.1, 1.9  B: 64.1, 1.6 | Knee osteoarthritis | (b) | Electroacupuncture | 5 weeks | 5 weeks | 𝛽-Endorphin |
| Karatay 2011 [19] | A: 17 (11:6)  B: 18 (12:6) | A: 21.29, 1.83  B: 22.39, 1.91 | Healthy young subject | (b) | Manual acupuncture | 2 weeks | 2 weeks | TNF-α, CRP |
| Karst 2003 [20] | A: 11 (5: 6)  B: 11 (5: 6) | A: 32.64, 8.77  B: 31.09, 8.34 | Healthy volunteers | (c) | Manual acupuncture | 4 weeks | 4 weeks | 𝛽-Endorphin, TNF-𝛽 |
| Kim 2021 [21] | A: 60 (0:60)  B: 60 (0:60) | A: 36.92, 7.93  B: 36.83, 8.79 | Premenopausal overweight and obese women | (b) | Electroacupuncture | 6 weeks | 6 weeks | TC, HDL, LDL. TG, FBS, Insulin, CRP |
| Kristen 2010 [22] | A: 8 (6:2)  B: 9 (5:4) | A: 58, 12  B: 60, 3 | Heart failure | (c) | Manual acupuncture | 5 weeks | 5 weeks | TNF-α |
| Kvorning 2010 [23] | A: 23  B: 22 | NR | Patients with scheduled surgery | (a) | Electroacupuncture | 1 day | 1 day | Adrenaline, Noradrenaline, ACTH, Cortisol |
| Lee 2015 [24] | A: 10  B: 10 | A+B 56.3, 10.5 | Cerebral infarction | (b) | Electroacupuncture | 1 day | 1 day | VEGF, IL-10 |
| Lee 2009 [25] | A: 12 (12:0)  B: 12 (12:0) | A: 36.4, 5.8  B: 23.5, 2.2 | Chronic prostatitis/Chronic pelvic pain syndrome | (b) | Electroacupuncture | 6 weeks | 6 weeks | NR |
| Leung 2011 [26] | A: 20 (8:12)  B: 20 (5:15) | A: 50.8, 5.8  B: 51.5, 4.6 | Healthy subject (Barostat-induced rectal distension) | (c) | Electroacupuncture | 1 day | 1 day | 𝛽-Endorphin |
| Li 2013 [27] | A: 19 (11:8)  B: 19 (10:9) | A: 39, 8  B: 42, 9 | Patients undergoing supratentorial craniotomy | (b) | Electroacupuncture | 1 day | 1 day | IL-10, IL-8 |
| Li 2017 [28] | A: 20 (12:8)  B: 20 (11:9) | A: 58, 12  B: 57, 11 | Acute pancreatitis | (b) | Electroacupuncture | 5 days | 5 days | TNF-α, IL-10 |
| Liang 2012 [29] | A: 50  B: 50 | NR | Severe multiple trauma patients | (b) | Manual acupuncture | 7 days | 7 days | NR |
| Lien 2012 [30] | A: 24 (0:24)  B: 24 (0:24) | A: 39.2, 11.6  B: 42.2, 12.4 | Obese women | (a) | Auricular acupuncture | 4 weeks | 4 weeks | Adiponectin, Insulin, Ghrelin, Leptin |
| Lu 2009 [31] | A: 9 (0:9)  B: 10 (0:10) | A: 50, 9.9  B: 50.8, 10.6 | Women undergoing chemotherapy due to ovarian cancer | (b) | Electroacupuncture | 3 weeks | 3 weeks | NR |
| Marie Lynning 2021 [32] | A: 21 (5:16)  B: 23 (8:15) | A: 46.8, 10.5  B: 46.5, 10.2 | Multiple sclerosis | (c) | Manual acupuncture | 4 weeks | 4 weeks | IFN-γ, IL-1, IL-12, IL-6, IL-8, TNF-α, IL-10 |
| McDonald 2016 [33] | A: 37  B: 36 | NR | Persistent allergic rhinitis | (b) | Manual acupuncture | 8 weeks | 12 weeks | IG-E |
| Mehta 2014 [34] | A: 52 (37: 15)  B: 47 (32: 15) | A: 63, 11  B: 64.9, 11 | Stable ischemic heart disease | (a) | Manual acupuncture | 12 weeks | 12 weeks | NR |
| Painovich 2012 [35] | A: 12 (0:12)  B: 12 (0:12) | A: 56.8, 6.5  B: 57.2, 5.2 | Menopause | (a) | Manual acupuncture | 12 weeks | 12 weeks | Cortisol |
| Swanson 2015 [36] | A: 13 (9:4)  B: 12 (8:4) | A: 47.69, 4.8  B: 47.33, 9.1 | HIV-infected adults | (c) | Manual acupuncture | 8 weeks | 8 weeks | CRP, TC, HDL, LDL, TG |
| Takahashi 2021 [37] | A: 3 (3:0)  B: 3 (3:0) | 20.1, 0.4 | Healthy male adult | (a) | Electroacupuncture | 2 weeks | 2 weeks | Aldosterone, Epinephrine, Norepinephrine |
| Wang 2008 [38] | A: 10 (8:2)  B: 9 (8:1) | A: 57.1, 9.9  B: 57.7, 7.4 | Gastroparesis | (b) | Electroacupuncture | 2 weeks | 2 weeks | FBS, PP2, Gastrin, Motilin |
| Wang 2016 [39] | A: 19 (9:10)  B: 20 (11:9) | A: 27.11, 1.16  B: 28, 1.10 | Healthy adults | (b) | Manual acupuncture | 1 day | 1 day | SP, VIP, NPY |
| Wang 2019 [40] | A: 15 (0:15)  B: 21 (0:21) | NR | polycystic ovarian syndrome | (b) | Electroacupuncture | 16 weeks | 16 weeks | Adrenaline, Noradrenaline, Serotonin, GABA |
| Xi 2021 [41] | A: 29 (13:16)  B: 29 (12:17) | A: 41, 12  B: 44, 12 | Chronic insomnia | (b) | Electroacupuncture | 4 weeks | 4 weeks | GABA |
| Xie 2017 [42] | A; 30 (5:25)  B: 30 (6:24) | A: 34, 7  B: 33, 6 | Obese patients | (b) | Manual acupuncture | 8 weeks | 8 weeks | Leptin |
| Xu 2015 [43] | A: 30 (11:19)  B: 30 (12:18) | A: 53.2, 12.1  B: 52.73, 14.7 | Functional constipation | (b) | Electroacupuncture | 8 weeks | 8 weeks | Serotonin |
| Yiiu 2016 [44] | A: 8  B: 9 | NR | Phonotraumatic vocal pathologies | (c) | Manual acupuncture | 1 day | 1 day | NR |
| Yu 2014 [45] | A: 20 (14:6)  B: 20 (12:8) | A: 56, 15  B: 56, 17 | Patients under surgery with etomidate anesthesia | (b) | Electroacupuncture | 1 day | 1 day | NR |
| Zhang 2013a [46] | A: 30 (0:30)  B: 30 (0:30) | A: 32, 3  B: 31, 5 | in vitro fertilization-embryo transfer (IVF-ET) transvaginal oocyte retrieval | (c) | Manual acupuncture | 1 day | 1 day | NR |
| Zhang 2020 [47] | A: 54 (36:18)  B: 81 (50:31) | NR | Gastric cancer | (b) | Manual acupuncture | 3 sessions | 4 weeks | IFN-γ, IL-4, IL-6, CRP |
| Zhang 2013b [48] | A: 34 (1:33)  B: 36 (11:25) | A: 48.2, 9.8  B: 46.3, 9.9 | Depression | (c) | Electroacupuncture | 3 weeks | 2 weeks | Serotonin |
| Zhao 2020 [49] | A: 33 (13:20)  B: 34 (11:23) | A: 45.16, 11.39  B: 43.87, 10.51 | Depression and insomnia | (c) | Manual acupuncture | 8 weeks | 8 weeks | NPY, Substance-p |
| Zhao 2018 [50] | A: 30 (14:16)  B: 30 (12:18) | A: 66.7, 3.84  B: 65.23, 4.03 | Patients under knee replacement surgery | (c) | Electroacupuncture | 1 day | 1 day | IL-1, TNF-α |
| Zuppa 2015 [51] | A: 24 (5:19)  B: 24 (5:19) | A: 65.75, 3.8  B: 67.13, 4.9 | Healthy aged adult | (b) | Manual acupuncture | 5 weeks | 5 weeks | BDNF |

Sham acupuncture: A/ Test acupuncture: B

Sham acupuncture types: three groups including skin stimulation with acupuncture needles or without acupuncture needles such as cocktail stick or tooth picks (a), superficial needling on acupuncture points or non-acupuncture points (b), sham device without penetration-Park, Streitburger, Takakura sham device (c).

BDNF: brain derived neurotrophic factor; CRP: C-reactive protein; FBS: fasting blood glucose; GABA: γ-aminobutyric acid; HDL: high- density lipoprotein cholesterol; IFN-γ: Interferon gamma; IL: interleukin; LDL: low-density lipoprotein cholesterol; NGF: nerve growth factor; NPY: Neuropeptide Y; NR: not reported; PP2: 2-hr postprandial blood glucose; SP: substance-P; TC: total cholesterol; TG: Triglycerides; TNF-α: tumor necrosis factor-α; VIP: vasoactive intestinal peptide

**Supplementary 3. References of the included studies**

[1] H. Abdi, P. Abbasi-Parizad, B. Zhao, M. Ghayour-Mobarhan, S. Tavallaie, A.A. Rahsepar, S.M. Parizadeh, M. Safariyan, M. Nemati, M. Mohammadi, M. Darbandi, S. Darbandi, G.A. Ferns, Effects of auricular acupuncture on anthropometric, lipid profile, inflammatory, and immunologic markers: a randomized controlled trial study, J Altern Complement Med 18(7) (2012) 668-77.

[2] H. Abdi, B. Zhao, M. Darbandi, M. Ghayour-Mobarhan, S. Tavallaie, A.A. Rahsepar, S.M. Parizadeh, M. Safariyan, M. Nemati, M. Mohammadi, P. Abbasi-Parizad, S. Darbandi, S. Akhlaghi, G.A. Ferns, The effects of body acupuncture on obesity: anthropometric parameters, lipid profile, and inflammatory and immunologic markers, ScientificWorldJournal 2012 (2012) 603539.

[3] S. Ahsin, S. Saleem, A.M. Bhatti, R.K. Iles, M. Aslam, Clinical and endocrinological changes after electro-acupuncture treatment in patients with osteoarthritis of the knee, Pain 147(1‐3) (2009) 60‐66.

[4] B.B. Arnetz, M. Berg, I. Anderzén, T. Lundeberg, E. Haker, A nonconventional approach to the treatment of "environmental illness", J Occup Environ Med 37(7) (1995) 838-44.

[5] Y. Aydoğmuş, M. Sunay, H. Arslan, A. Aydın, A.K. Adiloğlu, H. Şahin, Acupuncture versus solifenacin for treatment of overactive bladder and its correlation with urine nerve growth factor levels: a randomized, placebo-controlled clinical trial, Urologia internationalis 93(4) (2014) 437‐443.

[6] T. Bao, L. Cai, J.T. Giles, J. Gould, K. Tarpinian, K. Betts, M. Medeiros, S. Jeter, N. Tait, S. Chumsri, et al., A dual-center randomized controlled double blind trial assessing the effect of acupuncture in reducing musculoskeletal symptoms in breast cancer patients taking aromatase inhibitors, Breast cancer research and treatment 138(1) (2013) 167‐174.

[7] R. Brustin, M. Toledano, T. Geffen, R. Goona, M. Hochberg, B. Kreisberg, S. Murad, J. Pitcovski, Immune Modulation and Treatment of Human Papilloma Virus-Related Warts with Energetics of Living Systems Acupuncture, Medical acupuncture 29(3) (2017) 145‐154.

[8] M. Chassot, J.A. Dussan-Sarria, F.C. Sehn, A. Deitos, A. de Souza, R. Vercelino, I.L. Torres, F. Fregni, W. Caumo, Electroacupuncture analgesia is associated with increased serum brain-derived neurotrophic factor in chronic tension-type headache: a randomized, sham controlled, crossover trial, BMC Complement Altern Med 15 (2015) 144.

[9] T. Chen, K. Wang, J. Xu, W. Ma, J. Zhou, Electroacupuncture Reduces Postoperative Pain and Analgesic Consumption in Patients Undergoing Thoracic Surgery: a Randomized Study, Evidence-based complementary and alternative medicine 2016 (2016).

[10] A. Fitri, M. Abdullah, I. Rengganis, H. Mihardja, I.S. Baginda, A. Masthura, Acupoint-catgut embedment combined with medication does not decrease interleukin-6 levels serum in patients with gastroesophageal reflux disease: a randomized controlled clinical trial, Medical journal of indonesia 28(3) (2019) 241‐245.

[11] M. Haddad-Rodrigues, A.M. Spano Nakano, J. Stefanello, R.C. Campos Pereira Silveira, Acupuncture for anxiety in lactating mothers with preterm infants: a randomized controlled trial, Evidence-based complementary and alternative medicine 2013 (2013).

[12] X.C. Han, Q.Z. Xing, Y. Li, X. Dong, X.W. Ma, L. Zhang, Q.Q. Wang, [Clinical trial of electroacupuncture at Neimadian-point for cancer pain], Zhen Ci Yan Jiu 45(10) (2020) 845-50.

[13] H. Harbach, B. Moll, R.H. Boedeker, U. Vigelius-Rauch, H. Otto, J. Muehling, G. Hempelmann, P. Markart, Minimal immunoreactive plasma β-endorphin and decrease of cortisol at standard analgesia or different acupuncture techniques, European Journal of Anaesthesiology 24(4) (2007) 370-376.

[14] Y. Huang, J. Meng, B. Sun, T. Xiang, X. Zhou, B. Xu, Y. Wu, Z. Chen, S. Zhang, Acupuncture for serum uric acid in patients with asymptomatic hyperuricemia: a randomized, double-blind, placebo-controlled trial, International journal of cardiology 232 (2017) 227‐232.

[15] H. Ito, O. Yamada, Y. Kira, T. Tanaka, R. Matsuoka, The effects of auricular acupuncture on weight reduction and feeding-related cytokines: a pilot study, BMJ open gastroenterology 2(1) (2015) e000013.

[16] J.H. Jeon, J. Yoon, C.K. Cho, I.C. Jung, S. Kim, S.H. Lee, H.S. Yoo, Effect of acupuncture for radioactive-iodine-induced anorexia in thyroid cancer patients: a randomized, double-blinded, sham-controlled pilot study, Integr Cancer Ther 14(3) (2015) 221-30.

[17] S. Joos, B. Brinkhaus, C. Maluche, N. Maupai, R. Kohnen, N. Kraehmer, E.G. Hahn, D. Schuppan, Acupuncture and moxibustion in the treatment of active Crohn's disease: a randomized controlled study, Digestion 69(3) (2004) 131‐139.

[18] R.W. Jubb, E.S. Tukmachi, P.W. Jones, E. Dempsey, L. Waterhouse, S. Brailsford, A blinded randomised trial of acupuncture (manual and electroacupuncture) compared with a non-penetrating sham for the symptoms of osteoarthritis of the knee, Acupuncture in Medicine 26(2) (2008) 69-78.

[19] S. Karatay, F. Akcay, K. Yildirim, F.H. Erdem, F. Alp, Effects of some acupoints (Du-14, Li-11, St-36, and Sp-6) on serum TNF-alpha and hsCRP levels in healthy young subjects, Journal of alternative and complementary medicine (new york, N.Y.) 17(4) (2011) 347‐350.

[20] M. Karst, D. Scheinichen, T. Rueckert, T. Wagner, B. Wiese, S. Piepenbrock, M. Fink, Effect of acupuncture on the neutrophil respiratory burst: a placebo-controlled single-blinded study, Complement Ther Med 11(1) (2003) 4-10.

[21] K.-W. Kim, W.-C. Shin, M.S. Choi, J.-H. Cho, H.-J. Park, H.H. Yoo, M.-Y. Song, Effects of acupuncture on anthropometric and serum metabolic parameters in premenopausal overweight and obese women: a randomized, patient-and assessor-blind, sham-controlled clinical trial, Acupuncture in Medicine 39(1) (2021) 30-40.

[22] A.V. Kristen, B. Schuhmacher, K. Strych, D. Lossnitzer, H.C. Friederich, T. Hilbel, M. Haass, H.A. Katus, A. Schneider, K.M. Streitberger, et al., Acupuncture improves exercise tolerance of patients with heart failure: a placebo-controlled pilot study, Heart (British Cardiac Society) 96(17) (2010) 1396‐1400.

[23] N. Kvorning, J. Akeson, Plasma adrenaline increases in anesthetized patients given electro-acupuncture before surgery, Pain Med 11(7) (2010) 1126-31.

[24] S. Lee, W. Kim, J. Park, H.H. Jang, S.M. Lee, J.S. Woo, H.S. Kim, K.H. Lee, Y.J. Kwon, U. Lee, J.B. Kim, W.S. Kim, K.S. Kim, Effects of electroacupuncture on endothelial function and circulating endothelial progenitor cells in patients with cerebral infarction, Clin Exp Pharmacol Physiol 42(8) (2015) 822-7.

[25] S.H. Lee, B.C. Lee, Electroacupuncture relieves pain in men with chronic prostatitis/chronic pelvic pain syndrome: three-arm randomized trial, Urology 73(5) (2009) 1036-41.

[26] W.W. Leung, A.Y.M. Jones, S.S.M. Ng, C.Y.N. Wong, J.F.Y. Lee, Electroacupuncture in Reduction of Discomfort Associated with Barostat-Induced Rectal Distension-A Randomized Controlled Study, Journal of Gastrointestinal Surgery 15(4) (2011) 660-666.

[27] G. Li, S. Li, L. An, B. Wang, Electroacupuncture alleviates intraoperative immunosuppression in patients undergoing supratentorial craniotomy, Acupunct Med 31(1) (2013) 51-6.

[28] L. Li, J. Yu, R. Mu, S. Dong, Clinical effect of electroacupuncture on lung injury patients caused by severe acute pancreatitis, Evidence-Based Complementary and Alternative Medicine 2017 (2017).

[29] H. Liang, J. Qu, Decreased incidence of SIRS and sepsis by acupuncture in severe multiple traumatic patients via facilitation of vagal activity, Critical care (london, england) 16 (2012).

[30] C.Y. Lien, L.L. Liao, P. Chou, C.H. Hsu, Effects of auricular stimulation on obese women: a randomized, controlled clinical trial, European journal of integrative medicine 4(1) (2012) e45‐e53.

[31] W. Lu, U.A. Matulonis, A. Doherty-Gilman, H. Lee, E. Dean-Clower, A. Rosulek, C. Gibson, A. Goodman, R.B. Davis, J.E. Buring, P.M. Wayne, D.S. Rosenthal, R.T. Penson, Acupuncture for chemotherapy-induced neutropenia in patients with gynecologic malignancies: A pilot randomized, sham-controlled clinical trial, Journal of Alternative and Complementary Medicine 15(7) (2009) 745-753.

[32] M. Marie Lynning, 1 Kirsten Hanehøj, MS,1 Katrine Westergaard, MS,1 Annette Kjær Ersbøll, PhD,2 Mogens Helweg Claesson, DMSc,3 Finn Boesen, MD,4 and Lasse Skovgaard, PhD1,i, Effect of Acupuncture on Cytokine Levels in Persons with Multiple Sclerosis:

A Randomized Controlled Trial, THE JOURNAL OF ALTERNATIVE AND COMPLEMENTARY MEDICINE (2021).

[33] J.L. McDonald, P.K. Smith, C.A. Smith, C. Changli Xue, B. Golianu, A.W. Cripps, Effect of acupuncture on house dust mite specific IgE, substance P, and symptoms in persistent allergic rhinitis, Annals of allergy, asthma & immunology 116(6) (2016) 497‐505.

[34] P.K. Mehta, D.M. Polk, X. Zhang, N. Li, J. Painovich, K. Kothawade, J. Kirschner, Y. Qiao, X. Ma, Y.D. Chen, A. Brantman, C. Shufelt, M. Minissian, C.N. Merz, A randomized controlled trial of acupuncture in stable ischemic heart disease patients, Int J Cardiol 176(2) (2014) 367-74.

[35] J.M. Painovich, C.L. Shufelt, R. Azziz, Y. Yang, M.O. Goodarzi, G.D. Braunstein, B.Y. Karlan, P.M. Stewart, C.N. Merz, A pilot randomized, single-blind, placebo-controlled trial of traditional acupuncture for vasomotor symptoms and mechanistic pathways of menopause, Menopause 19(1) (2012) 54-61.

[36] B. Swanson, J.K. Keithley, A. Johnson, L. Fogg, O. Adeyemi, B.E. Sha, K.A. Snell, Acupuncture to reduce HIV-associated inflammation, Evidence-based complementary and alternative medicine 2015 (2015).

[37] H. Takahashi, T. Nabeta, H. Waki, K. Takeoka, H. Kawahata, N. Ideguchi, M. Aoki, T. Ogihara, Effect of Electroacupuncture on Stress-induced High Blood Pressure via Autonomic Nervous System in Normotensive Young Adults, Acupuncture & Electro-Therapeutics Research 45(2-3) (2021) 59-72.

[38] C.P. Wang, C.H. Kao, W.K. Chen, W.Y. Lo, C.L. Hsieh, A single-blinded, randomized pilot study evaluating effects of electroacupuncture in diabetic patients with symptoms suggestive of gastroparesis, J Altern Complement Med 14(7) (2008) 833-9.

[39] K. Wang, L. Chen, Y. Wang, C. Wang, L. Zhang, Sphenopalatine Ganglion Acupuncture Improves Nasal Ventilation and Modulates Autonomic Nervous Activity in Healthy Volunteers: A Randomized Controlled Study, Sci Rep 6 (2016) 29947.

[40] Z. Wang, H. Dong, Q. Wang, L. Zhang, X. Wu, Z. Zhou, L. Yang, D. Huang, Effects of electroacupuncture on anxiety and depression in unmarried patients with polycystic ovarian syndrome: secondary analysis of a pilot randomised controlled trial, Acupunct Med 37(1) (2019) 40-46.

[41] H.Q. Xi, W.Z. Wu, C.Y. Liu, X.Q. Wang, S. Qin, Y.N. Zhao, S.Y. Zheng, J.H. Li, Q.Y. Wan, Effect of acupuncture at Tiaoshen acupoints on hyperarousal state in chronic insomnia, Zhongguo zhen jiu [Chinese acupuncture & moxibustion] 41(3) (2021) 263‐267.

[42] X.C. Xie, Y.Q. Cao, Q. Gao, C. Wang, M. Li, S.G. Wei, Acupuncture Improves Intestinal Absorption of Iron in Iron-deficient Obese Patients: A Randomized Controlled Preliminary Trial, Chin Med J (Engl) 130(5) (2017) 508-515.

[43] H.F. Xu, C. Feng, H.X. Zhang, L. Zhou, Electro-acupuncture regulates plasma NOS and 5-HT levels in patients with severe functional constipation, World chinese journal of digestology 23(11) (2015) 1849‐1854.

[44] E.M. Yiu, K.M. Chan, N.Y. Li, R. Tsang, K. Verdolini Abbott, E. Kwong, E.P. Ma, F.W. Tse, Z. Lin, Wound-healing effect of acupuncture for treating phonotraumatic vocal pathologies: A cytokine study, Laryngoscope 126(1) (2016) E18-22.

[45] J.B. Yu, S.A. Dong, L.R. Gong, M. Wang, R. Mu, C. Li, Y. Zhang, Z.D. Li, Effect of electroacupuncture at Zusanli (ST36) and Sanyinjiao (SP6) acupoints on adrenocortical function in etomidate anesthesia patients, Med Sci Monit 20 (2014) 406-12.

[46] J. Zhang, X. Wang, R. Lu, Analgesic effect of acupuncture at Hegu (LI 4) on transvaginal oocyte retrieval with ultrasonography, Journal of traditional chinese medicine / chung i tsa chih ying wen pan 33(3) (2013) 294‐297.

[47] Y.J. Zhang, Q. Min, Y. Huang, H.D. Liu, Z.Y. Zhu, F.J. Jiang, H.Q. Hua, Efficacy of Acupuncture and Moxibustion as a Subsequent Treatment after Second-Line Chemotherapy in Advanced Gastric Cancer, Evidence-based Complementary and Alternative Medicine 2020 (2020).

[48] Z.J. Zhang, R. Ng, S.C. Man, J.T. Li, W. Wong, H.K. Wong, D. Wang, M.T. Wong, A.W. Tsang, K.C. Yip, S.C. Sze, Use of electroacupuncture to accelerate the antidepressant action of selective serotonin reuptake inhibitors: a single-blind, randomised, controlled study, Hong Kong Med J 19 Suppl 9 (2013) 12-6.

[49] F.Y. Zhao, Y. Xu, L.P. Yue, Y.X. Zhao, Y. Wang, H.L. Song, H. Xu, S.H. Chen, Q.Q. Fu, Manual acupuncture for patients with major depressive disorder and comorbid insomnia: Evidence from polysomnography and serum biomarkers, World Journal of Acupuncture - Moxibustion 30(1) (2020) 5-12.

[50] F.Y. Zhao, Z.Y. Zhang, Y.X. Zhao, H.X. Yan, Y.F. Hong, X.J. Xia, H. Xu, The effect of electroacupuncture preconditioning on cognitive impairments following knee replacement among elderly: a randomized controlled trial, World journal of acupuncture - moxibustion 28(4) (2018) 231‐236.

[51] C. Zuppa, C.H. Prado, A. Wieck, A. Zaparte, A. Barbosa, M.E. Bauer, Acupuncture for sleep quality, BDNF levels and immunosenescence: a randomized controlled study, Neuroscience letters 587 (2015) 35‐40.

**Supplementary file 4. Risk of bias assessment**

| **Study ID** | **Sequence generation** | **Allocation concealment** | **Blinding of participants** | **Blinding of outcome assessor** | **Incomplete outcome data** | **Selective outcome reporting** | **Other bias** | **Overall risk of bias** |
| --- | --- | --- | --- | --- | --- | --- | --- | --- |
| Abdi 2012a [1] | U | U | L | U | H | L | L | H |
| Abdi 2012b [2] | U | U | L | U | H | L | L | H |
| Ahsin 2009 [3] | H | H | L | L | H | L | L | H |
| Arnetz 1995 [4] | U | U | L | U | H | L | L | H |
| Aydoğmuş 2014 [5] | U | U | L | U | H | L | L | H |
| Bao 2013 [6] | L | U | L | L | L | L | L | L |
| Brustin 2017 [7] | U | U | L | U | L | L | L | H |
| Chassot 2015 [8] | L | L | L | L | L | L | L | L |
| Chen 2016 [9] | L | L | L | U | L | L | L | L |
| Fitri 2019 [10] | L | L | L | U | L | L | L | L |
| Haddad-Rodrigues [11] | L | L | L | L | H | L | L | H |
| Han 2020 [12] | L | L | L | U | L | L | L | L |
| Harbach 2007 [13] | L | L | L | L | L | L | L | L |
| Huang 2017 [14] | L | L | L | L | H | L | L | H |
| Ito 2015 [15] | L | L | L | L | L | L | L | L |
| Jeon 2015 [16] | L | L | L | L | L | L | L | L |
| Joos 2004 [17] | U | U | L | L | H | L | L | H |
| Jubb 2008 [18] | U | U | L | U | L | L | L | H |
| Karatay 2011 [19] | U | U | L | L | L | L | L | L |
| Karst 2003 [20] | U | U | L | L | L | L | L | L |
| Kim 2021 [21] | L | L | L | L | L | L | L | L |
| Kristen 2010 [22] | L | L | L | L | H | L | L | H |
| Kvorning 2010 [23] | U | U | L | L | L | L | L | L |
| Lee 2015 [24] | U | U | L | U | L | L | L | H |
| Lee 2009 [25] | L | U | L | L | L | L | L | L |
| Leung 2011 [26] | L | L | L | L | L | L | L | L |
| Li 2013 [27] | U | U | L | U | L | L | L | H |
| Li 2017 [28] | L | U | L | U | U | L | L | H |
| Liang 2012 [29] | U | U | L | U | U | L | L | H |
| Lien 2012 [30] | L | U | L | U | U | L | L | H |
| Lu 2009 [31] | L | U | L | L | H | L | L | H |
| Marie Lynning 2021 [32] | L | U | L | L | H | L | L | H |
| McDonald 2016 [33] | L | U | L | L | H | L | L | H |
| Mehta 2014 [34] | U | U | L | L | H | L | L | H |
| Painovich 2012 [35] | L | U | L | L | U | L | L | H |
| Swanson 2015 [36] | L | U | L | L | L | L | L | L |
| Takahashi 2021 [37] | U | U | L | L | L | L | L | H |
| Wang 2008 [38] | U | U | L | L | H | L | L | H |
| Wang 2016 [39] | L | L | L | L | L | L | L | L |
| Wang 2019 [40] | L | L | L | L | H | L | L | H |
| Xi 2021 [41] | U | U | L | L | L | L | L | H |
| Xie 2017 [42] | L | U | L | L | L | L | L | L |
| Xu 2015 [43] | U | U | L | L | L | L | L | H |
| Yiiu 2016 [44] | U | U | L | L | L | L | L | H |
| Yu 2014 [45] | L | U | L | L | L | L | L | L |
| Zhang 2013a [46] | U | U | L | L | L | L | L | H |
| Zhang 2020 [47] | L | L | L | L | H | L | L | H |
| Zhang 2013b [48] | U | U | L | L | H | L | L | H |
| Zhao 2020 [49] | L | U | L | L | L | L | L | L |
| Zhao 2018 [50] | L | L | L | L | L | L | L | L |
| Zuppa 2015 [51] | L | L | L | L | L | L | L | L |

L: low risk of bias; H: high risk of bias; U: unclear bias

**Supplementary file 5. Funnel plot**


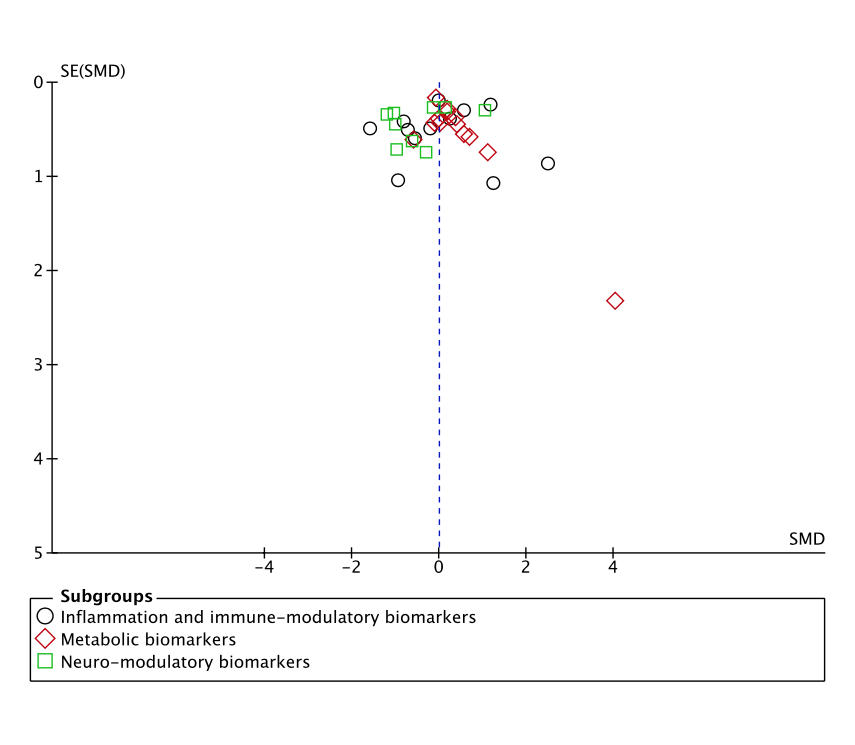

Supplement: Supplementary Table 1 — The correlation between clinical characteristics and subtypes. [file Table_1.DOCX]
