# Supplementary material for: Head and Neck Squamous Cell Carcinoma Subtypes Based on Immunologic and Hallmark Gene Sets in Tumor and Non-tumor Tissues
Source: Front Surg. 2022 Feb 3;9:821600. doi: 10.3389/fsurg.2022.821600 (PMC8850349; doi:10.3389/fsurg.2022.821600)
Supplement: Supplementary Table 2 — The details of 39 representative gene sets. [file Table_2.DOCX]

| id | HR | HR.95L | HR.95H | pvalue |
| --- | --- | --- | --- | --- |
| N_GSE22886_NAIVE_CD4_TCELL_VS_MONOCYTE_UP | 0.003791657 | 9.36E-05 | 0.153540055 | 0.003154782 |
| N_GSE16266_LPS_VS_HEATSHOCK_AND_LPS_STIM_MEF_UP | 0.004876903 | 0.000108351 | 0.219510364 | 0.006131691 |
| N_GSE16450_CTRL_VS_IFNA_6H_STIM_IMMATURE_NEURON_CELL_LINE_DN | 7.27E-05 | 2.63E-07 | 0.020118698 | 0.00089503 |
| T_GSE29618_BCELL_VS_PDC_UP | 0.005005132 | 0.000151316 | 0.165556281 | 0.003003242 |
| N_GSE22886_NAIVE_BCELL_VS_DC_UP | 0.005308342 | 0.000110213 | 0.255672491 | 0.008052334 |
| N_GSE1460_CD4_THYMOCYTE_VS_THYMIC_STROMAL_CELL_UP | 1.22E-06 | 4.34E-10 | 0.003454579 | 0.000784427 |
| N_GSE6566_STRONG_VS_WEAK_DC_STIMULATED_CD4_TCELL_DN | 1.91E-05 | 1.28E-08 | 0.028514074 | 0.003566917 |
| N_GSE22886_NAIVE_BCELL_VS_MONOCYTE_UP | 0.020009589 | 0.000968365 | 0.413463758 | 0.011355488 |
| N_GSE26495_PD1HIGH_VS_PD1LOW_CD8_TCELL_UP | 0.003551418 | 2.50E-05 | 0.505208644 | 0.025754214 |
| N_GSE36392_MAC_VS_NEUTROPHIL_IL25_TREATED_LUNG_UP | 5.04E-07 | 9.93E-11 | 0.002561181 | 0.000866386 |
| N_GSE24210_CTRL_VS_IL35_TREATED_TCONV_CD4_TCELL_UP | 0.002109364 | 9.37E-06 | 0.474668542 | 0.025773593 |
| N_GSE7219_UNSTIM_VS_LPS_AND_ANTI_CD40_STIM_NIK_NFKB2_KO_DC_UP | 0.00648789 | 9.19E-05 | 0.458260973 | 0.020384904 |
| T_GSE32901_NAIVE_VS_TH17_NEG_CD4_TCELL_UP | 0.007003138 | 0.00012115 | 0.404819005 | 0.016537326 |
| T_GSE12003_4D_VS_8D_CULTURE_MIR223_KO_BM_PROGENITOR_DN | 1.57E-06 | 5.30E-10 | 0.004642612 | 0.001047732 |
| T_HALLMARK_ALLOGRAFT_REJECTION | 0.068716247 | 0.006571045 | 0.718595342 | 0.025359007 |
| T_GSE40666_UNTREATED_VS_IFNA_STIM_STAT4_KO_EFFECTOR_CD8_TCELL_90MIN_UP | 0.000848016 | 7.25E-07 | 0.991311315 | 0.049717642 |
| N_GSE16450_IMMATURE_VS_MATURE_NEURON_CELL_LINE_12H_IFNA_STIM_UP | 0.003753221 | 3.52E-05 | 0.400613371 | 0.019085854 |
| N_GSE3982_MAST_CELL_VS_EFF_MEMORY_CD4_TCELL_DN | 0.001784248 | 8.28E-06 | 0.384614897 | 0.020971333 |
| N_GSE17974_IL4_AND_ANTI_IL12_VS_UNTREATED_4H_ACT_CD4_TCELL_UP | 9.10E-06 | 1.62E-09 | 0.051205385 | 0.008426296 |
| N_GSE28408_LY6G_POS_VS_NEG_DC_UP | 2.08E-05 | 8.55E-09 | 0.050487596 | 0.006714215 |
| N_GSE7219_UNSTIM_VS_LPS_AND_ANTI_CD40_STIM_DC_UP | 0.005421488 | 7.20E-05 | 0.408212995 | 0.017965605 |
| N_GSE22886_NAIVE_CD4_TCELL_VS_12H_ACT_TH1_UP | 1.97E-05 | 5.65E-09 | 0.068632062 | 0.009220929 |
| N_GSE22886_UNSTIM_VS_IL2_STIM_NKCELL_UP | 0.000134316 | 1.45E-07 | 0.12420764 | 0.010510784 |
| N_GSE14415_INDUCED_TREG_VS_FOXP3_KO_INDUCED_TREG_IL2_CULTURE_DN | 0.002012683 | 7.41E-06 | 0.546503937 | 0.02990992 |
| T_GSE29614_DAY3_VS_DAY7_TIV_FLU_VACCINE_PBMC_DN | 0.004085885 | 4.36E-05 | 0.382583337 | 0.017558036 |
| T_GSE45365_NK_CELL_VS_BCELL_MCMV_INFECTION_DN | 0.000264023 | 1.35E-07 | 0.51656937 | 0.033106799 |
| N_GSE22886_UNSTIM_VS_IL15_STIM_NKCELL_UP | 0.000503057 | 1.14E-06 | 0.221467441 | 0.014471806 |
| N_GSE40666_UNTREATED_VS_IFNA_STIM_STAT1_KO_CD8_TCELL_90MIN_DN | 0.010919921 | 0.000120387 | 0.990513346 | 0.049517503 |
| T_GSE29617_CTRL_VS_DAY7_TIV_FLU_VACCINE_PBMC_2008_DN | 4.55E-05 | 1.51E-08 | 0.137019215 | 0.014431213 |
| N_GSE43863_TH1_VS_TFH_EFFECTOR_CD4_TCELL_DN | 0.000722082 | 6.96E-07 | 0.749271425 | 0.041208206 |
| N_GSE3039_ALPHABETA_CD8_TCELL_VS_B1_BCELL_UP | 0.002033454 | 4.54E-06 | 0.910329058 | 0.046576591 |
| N_GSE22886_CD8_VS_CD4_NAIVE_TCELL_DN | 8.93E-05 | 7.90E-08 | 0.100969645 | 0.009344084 |
| N_GSE22886_NAIVE_CD4_TCELL_VS_12H_ACT_TH2_UP | 5.79E-05 | 2.44E-08 | 0.137136874 | 0.0138501 |
| N_GSE10325_CD4_TCELL_VS_LUPUS_CD4_TCELL_UP | 1.01E-05 | 1.42E-09 | 0.071792092 | 0.011022958 |
| N_GSE7460_WT_VS_FOXP3_HET_ACT_WITH_TGFB_TCONV_UP | 3.67E-05 | 1.15E-08 | 0.117414737 | 0.013132305 |
| N_GSE21033_3H_VS_24H_POLYIC_STIM_DC_UP | 0.001009293 | 4.47E-06 | 0.227693936 | 0.012588937 |
| N_GSE13547_WT_VS_ZFX_KO_BCELL_ANTI_IGM_STIM_12H_DN | 0.000452761 | 4.15E-07 | 0.493583246 | 0.030941404 |
| N_GSE17974_0H_VS_48H_IN_VITRO_ACT_CD4_TCELL_UP | 0.002910066 | 8.62E-06 | 0.982579652 | 0.049312453 |
| N_GSE22229_RENAL_TRANSPLANT_VS_HEALTHY_PBMC_DN | 0.000215592 | 1.58E-07 | 0.294630257 | 0.021923131 |
